# Analyzing Surgical Treatment of Intestinal Obstruction in Children with Artificial Intelligence

**DOI:** 10.1155/2021/6652288

**Published:** 2021-01-11

**Authors:** Wang-Ren Qiu, Gang Chen, Jin Wu, Jun Lei, Lei Xu, Shou-Hua Zhang

**Affiliations:** ^1^Computer Department, Jing-De-Zhen Ceramic Institute, Jing-De-Zhen 333046, China; ^2^School of Management, Shenzhen Polytechnic, Shenzhen 518000, China; ^3^Department of General Surgery, Jiangxi Provincial Children's Hospital, Nanchang, Jiangxi 330006, China; ^4^School of Electronic and Communication Engineering, Shenzhen Polytechnic, Shenzhen 518000, China

## Abstract

Intestinal obstruction is a common surgical emergency in children. However, it is challenging to seek appropriate treatment for childhood ileus since many diagnostic measures suitable for adults are not applicable to children. The rapid development of machine learning has spurred much interest in its application to medical imaging problems but little in medical text mining. In this paper, a two-layer model based on text data such as routine blood count and urine tests is proposed to provide guidance on the diagnosis and assist in clinical decision-making. The samples of this study were 526 children with intestinal obstruction. Firstly, the samples were divided into two groups according to whether they had intestinal obstruction surgery, and then, the surgery group was divided into two groups according to whether the intestinal tube was necrotic. Specifically, we combined 63 physiological indexes of each child with their corresponding label and fed them into a deep learning neural network which contains multiple fully connected layers. Subsequently, the corresponding value was obtained by activation function. The 5-fold cross-validation was performed in the first layer and demonstrated a mean accuracy (Acc) of 80.04%, and the corresponding sensitivity (Se), specificity (Sp), and MCC were 67.48%, 87.46%, and 0.57, respectively. Additionally, the second layer can also reach an accuracy of 70.4%. This study shows that the proposed algorithm has direct meaning to processing of clinical text data of childhood ileus.

## 1. Introduction

Intestinal obstruction is one of the most common surgical emergencies in children [1]. Doctors have difficulty making a diagnosis due to the complexity of the disease while a right choice would be helpful for making correct therapeutic schemes. Clinically, conservative treatment will be taken when the patients' intestine is only partially blocked. They can be cured by medical treatment. Children with their intestine completely blocked need timely surgery to prevent further deterioration.

There are some existing methods to combine artificial intelligence technology and medical data to build a computer-aided diagnosis (CAD) system and provide a computerized “second-opinion” to doctors in their diagnosis. Deep learning has taken an important role in related processing in many medical fields in recent years, but in most cases, it extracts data from medical images. For example, Xu et al. [2] combined wavelet transform and convolutional neural network to identify atrial fibrillation according to the frequency. Norgeot et al. [3] proposed a deep learning model using electronic medical record data to forecast complex disease outcomes. Levine et al. [4] described various applications of deep learning in cancer diagnosis in their review. Thus, deep learning plays an increasingly important role in medical diagnosis.

Although deep learning has good application in medical image data. However, when medical image data are involved in the topic, a lot of preparatory work is needed, for example, distinguishing the normal medical image samples, image calibration, feature calibration, and feature extraction [5]. Researchers have to repeat the complex work when the recognition result is not ideal, making the effort expended multiplied. Here, we try to extract information from the medical diagnostic texts instead of the medical image which is inconvenient for medical researchers. It is well known that when lesions develop in certain parts, some physiological indexes would be abnormal at the same time, and symptoms usually appear after the indexes change to a certain extent. If doctors can detect abnormalities before they occur, it may help to prevent the disease from getting worse. It can also provide meaningful diagnosis and treatment suggestions to help the doctor make diagnostic decisions when the disease occurs. Applying deep learning algorithms to text data can not only verify each other with models applied to medical image data but also improve the overall credibility of the model.

Many diseases cannot be considered a single disease; they even have many subtypes. Without the help of computers, it would be difficult for clinicians to diagnose these patients [6, 7]. In order to make up for the real-time changes of the disease in the process of diagnosis and treatment, Simon et al. [8] constructed the Oncology Expert Advisor (OEA) to provide patients with relevant consultation services, including patient history summary, treatment proposal, and management consultation. In 2019, Liu et al. [9] constructed an auxiliary diagnostic system for diagnosing diabetic circulatory complications which combined deep learning with some physiological parameters of the human body. It occurred to us whether deep learning can be combined with other text data and applied to the diagnosis of other diseases [10]. Taking this as a reference, deep learning is applied to the medical text data for diagnosis of intestinal obstruction in children, and an appropriate model is established to predict the condition of patients.

In this experiment, age, blood routine, liver and kidney function, and coagulation function of these children with intestinal obstruction were taken into consideration. These indexes are available in most hospitals, and values can be obtained in a short time by using armamentarium. The reason for using so many parameters is that the detection methods are different; in fact, the more data, the more accurate the conclusion for doctors. Compared with using a single inspection indicator, the results of multiple inspection parameters are more comprehensive and convincing. In addition, for patients with intestinal obstruction, these blood routine and some indicators (such as liver and kidney function) can directly reflect the actual situation. These index data were used to train a deep neural network with multiple hidden layers. However, incomplete and irregular data is the first problem that needs to be solved. Improper data processing can significantly impact model performance. Actually, when the data have vacancies, some researchers discard the incomplete data and only keep the complete samples, and others may fill the null values with parameters of similar data [11–14]. In this study, due to the high proportion of samples with missing values, it is unreasonable to remove those with missing values. Thus, the means of each category are used to fill the vacant data alone with the mode and median. But the latter are not as good as the former one. In addition, when it comes to nonnumeric data, some conversion rules are taken to process the data. After processing the sample through the input layer and then through the hidden layer of the deep neural network (mainly composed of several fully connected layers), the number of the nodes in the fully connected layer is reduced by half per layer. Finally, the results are given by the activation function of the output layer [15].

**Figure 1:**
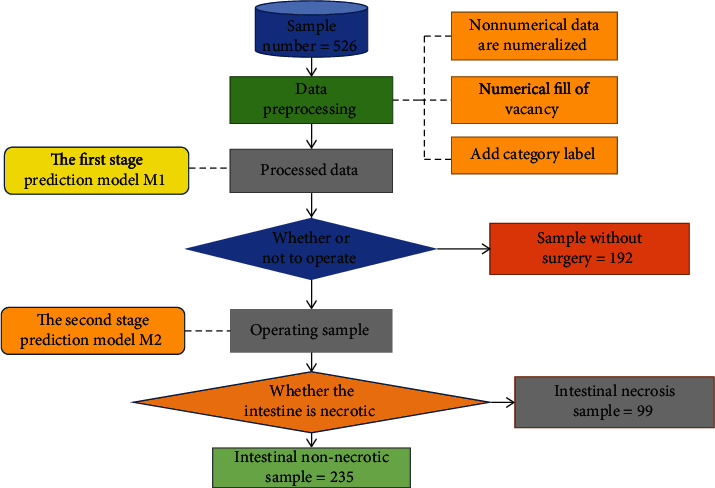
Experimental flow chart.

The purpose why we constructed such an auxiliary diagnostic system is to extract more useful information from the biochemical data of these children and determine whether they need surgery. The experimental flow chart is shown in Figure 1. Although the amount of data is not large, the results of the findings were pretty suggestive, which leads us to believe that as the amount of data increase, the performance of the model will get better. Secondly, the model has good generalization capability. With similar textual medical data, the model can be applied to predict or diagnose other diseases by changing certain parameters. The results also prove that deep learning is promising in the processing of text data, and the model has good application prospects.

## 2. Method

The main method used in this study is a deep neural network, which inputs all features into several hidden layer network structures composed of a full connection layer and finally classifies samples through classification function [16]. In order to compare the performance of the algorithm, the same feature data is also tested with other machine algorithms, such as support vector machine (SVM) and random forests (RF).

This retrospective study was approved by the ethics committee of Jiangxi Provincial Children's Hospital. And the participants were under the age of 18, so written informed consent was obtained from their guardian or legal next of kin. In this study, the children with intestinal obstruction hospitalized in Jiangxi Provincial Children's Hospital from January 2014 to June 2019 were selected as subjects, and the patients with shock, DIC, tumor, and abdominal surgery within 30 days were excluded from this study.

### 2.1. Deep Neural Network (DNN)

Since the dimension of data features is an acceptable range, this experiment constructs the learning model by adding several layers of full connection layers to the hidden layer of the neural network. In this method, the input value of each layer is passed through the output value generated by a specific filter and then input to the filter of the next layer [17–19]. The principle of the filter is shown in

(1) $$\begin{array}{c} R=\sigma \left(\overset{l}{\underset{k=1}{\sum }}w_{k}x_{t+k}+b\right). \end{array}$$

In formula (1), *R* is the output value of the filter, *σ* is the activation function, *w*_*k*_ is the *k*th weight matrix, *x*_*t*+*k*_ is the (*t* + *k*)th input value, and *b* is the bias value of the entire filter.

### 2.2. Support Vector Machine (SVM)

Support vector machines (SVM) are generally used to solve classification problems in two cases, linearly separable and linearly indivisible. Similarly, two points lower than two dimensions can be linearly separable. However, for high-dimensional data, it is often difficult to judge their boundaries. In this case, it is necessary to map their dimensions to higher dimensions so that its hyperplane can be found [20, 21]. The principle involved is shown in

(2) $$\begin{array}{c} f\left(x\right)=\mathrm{sign}\left(\overset{M}{\underset{l=1}{\sum }}\alpha_{l}K\left(x,j_{l}\right)+b\right). \end{array}$$

In formula (2), *f*(*x*) is the final decision value, sign is the classification decision function, *α*_*l*_ is a coefficient vector, *K* is the activation function, and *j*_*l*_ is the input training vector.

### 2.3. Random Forests (RF)

Random forests are formed based on decision trees. The difference from decision trees is that random forests use a combination of set ideas and bagging integration methods. The self-service method is used to randomly select *X* samples from the total number of samples to form a decision tree and then conduct *Y* times in total. In this way, we can construct *Y* decision trees. The final vote between these decision trees determines the outcome of the random forest [22]. In this way, the learning ability of weak learners can be improved by integrating ideas.

## 3. Experiment Design

### 3.1. Datasets

The sample data used in this experiment were collected from the diagnosis data of intestinal obstruction in Jiangxi Children's Hospital from January 2014 to June 2019, including 526 cases [23] which are listed in the supplementary file (available here). The diagnostic data included 64 parameters such as age, sex, blood routine, liver and kidney function, and coagulation function. The names of all parameters are listed in Table 1. According to the actual diagnosis and treatment of patients, corresponding label attributes were added for each patient. Of the 192 patients, those who did not receive surgical treatment for ileus but received conservative treatment were labeled K1. The other 235 patients who underwent surgery for ileus but had no intestinal necrosis were labeled K2, and the last 99 patients who underwent surgery for ileus and had intestinal necrosis were labeled K3.

To more accurately judge the patient's physical condition and timely make the correct diagnosis, the samples were grouped into positive samples and negative samples. It can be expressed as

(3) $$\begin{array}{c} S=S^{+}\cup S^{-}. \end{array}$$

Among them, *S* represents the total number of 526 cases of ileus, *S*^+^ represents the positive sample of 192 patients who received conservative treatment without surgery, and *S*^−^ represents the negative sample of 334 patients who underwent surgery. To further determine whether the intestinal tract is necrotic in the patient undergoing intestinal obstruction surgery, negative samples were divided into two groups in the experiment, i.e., *S*^−^ were expressed as

(4) $$\begin{array}{c} S^{-}=S_{g}^{-}\cup S_{b}^{-}. \end{array}$$

*S*_*g*_^−^ represents 235 patients who underwent surgery but had no intestinal necrosis and *S*_*b*_^−^ represents 99 patients who underwent surgery and had intestinal necrosis, as shown in Table 2.

**Figure 2:**
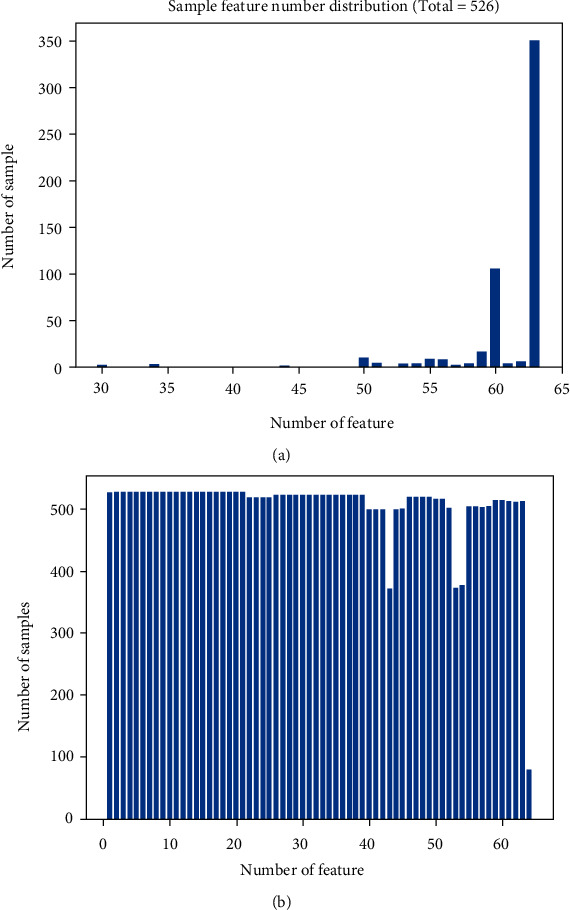
Profile of the benchmark.

The characteristic parameters of these samples include digital data and nonnumerical data. Numeric parameters are used directly. For nonnumerical data, this experiment adopted a quantitative method to convert these data. For example, the patient's age attribute value was 2 years and 8 months, and the converted value was 2.67.  Figure 2(a) shows the integrity information between the total sample number and the number of characteristic parameters. Of the 526 samples, only 59, 11.2 percent of the total benchmark, did not miss any feature(s). Deleting the incomplete samples will definitely result in a lot of information being discarded, greatly reducing the number of samples. Obviously, it is unreasonable to discard the samples with missing value(s) in this experiment. Therefore, the experiment decided to use the retention method to fill in the missing values. In this way, the incomplete samples can reach the dimension of the normal sample data and can be put into the model for training. However, Figure 2(b) shows the last one-dimensional characteristic (amylase property) of many samples missing the feature. To prevent the filling element from interfering too much with the original sample values, we decided to abandon this feature. In the deep learning algorithm, the larger the feature dimension of the sample is, the more information can be mined. Therefore, 63-dimensional characteristics other than amylase were used in this study. Whether there is a statistical error in the sample data has not been discussed here. We can even use these statistical error data served for model scalability tests.

Figure 2(a) shows the histogram of the dimensional distribution of the sample features. Figure 2(b) shows the histogram of sample quantity distribution with a certain characteristic.

### 3.2. Data Filling

In order to make the missing data closer to the actual situation of the patients, the category-mean method was adopted in this experiment to fill in the blank of the sample. The specific principle is shown in Equations (5) and (6).

First, we divided all the sample data into male and female categories based on gender attributes. Secondly, according to the above three categories, the data of each gender is divided into three categories. Then, the average of each attribute of each of the six categories was taken in turn. Finally, the blanks were filled in with the corresponding average:

(5) $$\begin{array}{c} \text{Mea}{\text{n}}_{i}=\frac{1}{m}\left[\overset{m}{\underset{j=1}{\sum }}x_{j1},\overset{m}{\underset{j=1}{\sum }}x_{j2},\cdots ,\overset{m}{\underset{j=1}{\sum }}x_{j63}\right],\;i=1,2,3,4,5,6, \end{array}$$

(6) $$\begin{array}{c} N_{pq}=\text{Mea}{\text{n}}_{i}\left[q\right]. \end{array}$$

Mean_*i*_ represents the mean matrix of 63 parameters for each classification, *x*_*j*1_, ⋯, *x*_*j*63_ represents the 63 eigenvalues of the *j*th sample, *m* is the number of samples for each of the six categories, and *N*_*pq*_ is the value of the *i*th class and the *q*th eigenvalue.

### 3.3. Model Design and Training

**Figure 3:**
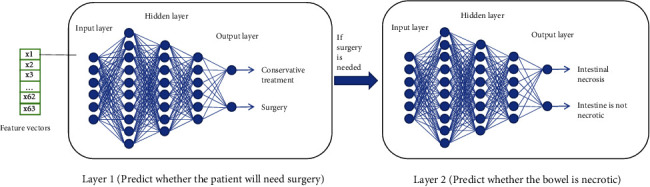
Two layers of deep learning neural network.

To build the first layer model, we used 420 samples from the processed sample data as the training dataset and the remaining 106 samples as the independent testing dataset [24]. In order to verify the stability of the model, a 5-fold cross-validation method is adopted to further verify the model. The 5-fold cross-validation refers to dividing 420 processed samples into 5 groups with almost equal proportions of categories between each group. The performance of these five models is compared to reflect the overall stability of the model [25]. The hidden layer of the deep neural network model used in this experiment is mainly composed of several completely connected layers. The final output is determined by the forward propagation algorithm of DNN. A second layer model was established on the basis of further examination of 334 patients undergoing intestinal obstruction surgery [26]. This layer model is used to identify intestinal necrosis in patients with intestinal obstruction requiring surgery. The structure of the second layer model is basically the same as that of the first layer model. The principle of the deep learning model is shown in Figure 3.

In order to make it easier to remember and use, the model of the first layer is M1 and the model of the second layer is M2 in this experiment. The purpose of M1 was to analyze whether patients needed to be treated for ileus. At the same time, we should consider the priority between ileus operations. If the patient's condition is at risk, the doctor should immediately arrange for an operation. If the patient's condition is not serious, the condition should be observed first, and then, appropriate treatment should be taken. The objective of M2 is to analyze the patient's condition priority and determine whether there is intestinal necrosis in patients requiring surgery. The longer the patient waits in an emergency, the more dangerous the patient's condition becomes. Therefore, the establishment of a two-layer prediction model can reasonably and objectively analyze the specific conditions of patients with intestinal obstruction and provide more accurate diagnostic results for doctors.

This intelligent assistant diagnostic model system can help doctors make decisions. To some extent, the model can reduce the possibility of misdiagnosis and make good progress in conservative treatment. In order to objectively evaluate the performance of the model, the potential correlation between features is explored. Then, we establish the random forest algorithm and support vector machine algorithm model.

In the deep learning model, all available eigenvalues are input into the neural network for training. The results provided by the model reflect the overall likelihood of the patient, but the contribution of these characteristic values to the model is not clear. Therefore, the Gini coefficient or information entropy is also needed to evaluate the importance of features. This experiment ranks the importance of all features by using the feature importance method in Python's Sklearn library [27]. The feature importance method ranks the features according to the number of Gini coefficient drops. Specifically, a feature is first selected, and then, the sum of the decline degree of Gini index of branch nodes formed by the feature in each tree of the random forest is counted. The sum value is the importance score of the feature. We select the 20 most important features which are listed in Table 3 and the variances and mean values of the 10 most important features which are listed in Table 4.

From the importance score of overall features, it can be seen that the contribution of individual features in the model is not high. The maximum importance score was less than 0.05, and the minimum value was only 0.003. It can be seen that the contribution of a single feature to the model is not significant enough. As shown in Table 3, the importance scores of creatinine isoenzyme, serum amyloid A, creatinine, and C-reactive protein were all higher than 0.035. Compared with other features, these four features are of great importance. It was evident from the sample data that C-reactive protein levels were higher in these patients with intestinal necrosis than in those receiving conservative treatment. This may be due to severe infection in necrotic parts of the intestine, which promotes the production of C-reactive protein and keeps the C-reactive protein content at a high level. Therefore, C-reactive protein can be used as an important reference parameter for current treatment decisions.

## 4. Result and Discussion

### 4.1. Performance Evaluations

In this experiment, four indicators were used to evaluate the performance of the model: accuracy (Acc), sensitivity (Se), specificity (Sp), and Matthews correlation coefficient (MCC) [28]. The total number of positive samples correctly predicted as positive samples was represented by TP, the total number of positive samples wrongly predicted as negative samples was represented by FN, the total number of negative samples correctly predicted as negative samples was represented by TN, and the total number of negative samples wrongly predicted as positive samples was represented by FP. The relationship between the four indicators and parameters is listed as follows:

(7) $$\begin{array}{c} \text{Acc}=\frac{\text{TP}+\text{TN}}{\text{TP}+\text{FP}+\text{TN}+\text{FN}}, \end{array}$$

(8) $$\begin{array}{c} \text{Se}=\frac{\text{TP}}{\text{TP}+\text{FN}}, \end{array}$$

(9) $$\begin{array}{c} \text{Sp}=\frac{\text{TN}}{\text{TN}+\text{FP}}, \end{array}$$

(10) $$\begin{array}{c} \text{MCC}=\frac{\text{TP}*\text{TN}-\text{FP}*\text{FN}}{\sqrt{\left(\text{TN}+\text{FN}\right)*\left(\text{TN}+\text{FP}\right)*\left(\text{TP}+\text{FN}\right)*\left(\text{TP}+\text{FP}\right)}}. \end{array}$$

**Figure 4:**
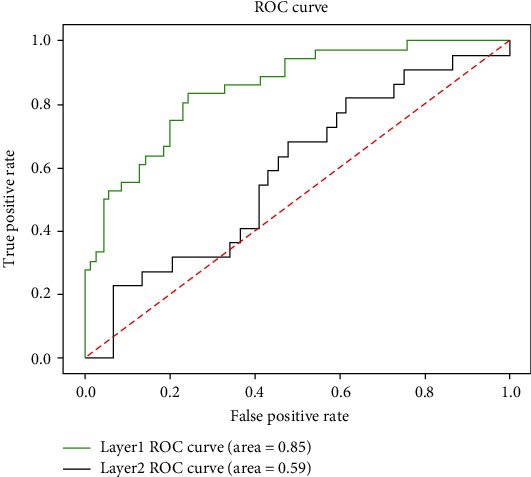
ROC curves of M1 and M2.

The final results of model 5-fold cross-validation are shown in Table 5. As can be seen from Table 5, M1 is used to diagnose whether a patient needs surgery. The four indicators of model M1 are, respectively, the following: Acc is 80.04%, Se is 67.48%, Sp is 87.46%, and MCC is 0.57. M2 is used to further diagnose intestinal necrosis in patients with intestinal obstruction. The four indicators of M2 are, respectively, the following: Acc is 66.78%, Se is 13.16%, Sp is 90.15%, and MCC is 0.18. In addition to representing the performance of the model with MCC, we also drew the ROC curve of the two-layer model. The area of a ROC curve, also known as the AUC value, is usually between 0 and 1. The larger the AUC value is, the better the model performance will be. The ROC curve is shown in Figure 4.

### 4.2. Discussion

In this paper, an intelligent auxiliary diagnostic system is proposed based on blood biochemical parameters and a deep learning algorithm. The aim of this study is to provide the decision of the next treatment for children with ileus. Secondly, the performance of the aided intelligent diagnostic system is compared by training different machine learning models [29].  Table 6 shows the performance of these different models trained in the first layer using the same processing data.

It can be seen from Table 6 that the performance of the support vector machine in the first layer model is lower than the training results of the other two models. The reason may be the size of these biochemical parameters. After the normalization of these eigenvalues, the discriminability of feature categories will be greatly reduced. Finally, the performance of the model will be greatly affected after these normalized data are input into the SVM. Therefore, the support vector machine algorithm should be avoided when the magnitude difference of text data is large. The performance of the random forest algorithm is close to that of the deep learning model, mainly because the swarm intelligence learning of the random forest plays an important role. Random forest is a kind of integration algorithm. By integrating decision trees with weak classification performance, the error of each decision tree can be averaged and the performance of the model can be improved as a whole.

The M1 model was used to diagnose whether patients with intestinal obstruction need further surgical models. Good predictive results can be obtained simply by using blood routine fluid, liver and kidney function, coagulation function, and other indicators. Compared with other medical image data, medical text data is also very important for the development of diagnosis, and these medical text data can be easily and economically obtained by examination in many hospitals. With the development of medical text data architecture, the performance of this model will be improved continuously.

The M2 model was used to further investigate whether the intestinal tract was necrotic in patients with ileus. As you can see from Table 5, the Se mean of M2 is very low, only 13.16%. The reasons for misclassification may be complicated clinical symptoms, limited blood biochemical indexes, or small sample size of model training. Finally, the M2 model makes it difficult to distinguish between intestinal necrosis and nonintestinal necrosis in ileus patients. Therefore, misdiagnosis may occur when text data is used alone. Of course, we cannot completely deny the importance of medical textual data. We can combine medical image data with models built from medical text data to make diagnostic decisions for patients. The medical text features used in this experiment may contain more noise features. Subsequent studies can filter out those irrelevant noise features in advance to improve the performance of the model. Secondly, in addition to these characteristic parameters, some types of parameters (such as blood type) are not used. In order to improve the performance of the diagnostic system, future research work may add these types of parameters to the general decision through some coding methods.

Although the parameters used in this study were 63 dimensions, they were very limited in terms of disease prediction. Symptoms of the patients were not used in this study. Compared to other parameters, the symptom characteristics reflect the patient's condition well. In the future, we plan to use more combinatorial experiments in feature parameter selection. The model of the algorithm is further optimized to improve the performance of models.

## 5. Conclusion

**Figure 5:**
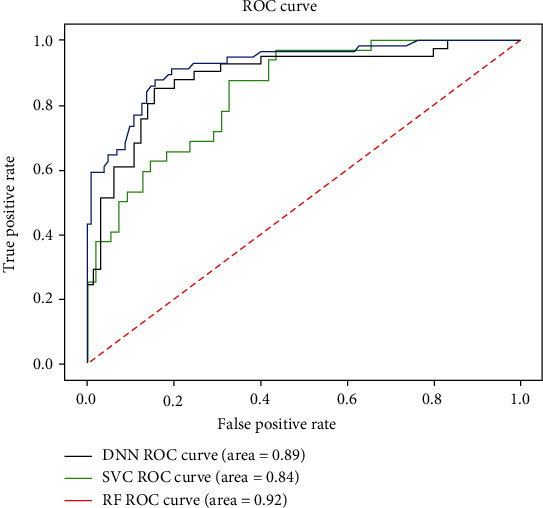
ROC graph of each algorithm.

The present model proposes a deep learning model for predicting the treatment of intestinal obstruction in children [30], by establishing some related learning models to further evaluate the importance of each function and initially obtaining the impact indicators that need attention. Based on blood and other medical data, an auxiliary intelligent diagnosis system was established. The purpose is to reduce the risk of misdiagnosis and provide doctors with supportive decisions. The ROC curves of these three algorithms are shown in Figure 5. It can be seen that the AUC value of the support vector machine is lower than that of the other two algorithms. The reason why the AUC value of the deep neural network is smaller than the random forest may be because the training samples are too small. The authors believe that by accumulating data and sample size, the performance of deep neural network models will continue to improve.

With the continuous updating of medical equipment and medical diagnostic data related to explosive growth, if the intelligent diagnostic algorithm is limited to medical image data, the result is actually incomplete. Usually, only certain clinical features can detect a disease, and then, doctors will take appropriate diagnostic measures. But these are not enough to control or prevent disease. Before some diseases appear in the human body, some biochemical parameters of the body first appear abnormal. Therefore, the hidden information of these biochemical parameters can be dug out. Early warning is given before the disease arrives, and many diseases will be controlled in advance. Although this research is only a small step, only through deep learning combined with a large number of examples in medical text data to help patients diagnose can the model be continuously improved. With the development of information technology, more and more methods are applied to medical text data, and the model will be more accurate and efficient.

In the experiment, we found that the mode and median sequence were used to process the empty data, and the processed data had great differences in model performance. Of course, compared with the other two methods, the filling method used in this article is the best. Therefore, in order to avoid model overfitting, which leads to poor model generalization, it is necessary to conduct reasonable screening only from the aspect of filling data. In the future, we will add parameter information not used in this experiment, integrate it into other feature parameters to build feature vectors, and try to find some key biochemical parameters important to the disease through the convolutional neural network [31].

## Figures and Tables

**Table 1:** Clinical parameters.

Clinical parameter name
Activated partial thromboplastin time, age, alkaline phosphatase, alanine aminotransferase (ALT), albumin, albumin to globulin ratio, amylase, aspartate aminotransferase (AST), AST/ALT, blood urea nitrogen (BUN), BUN/CR, calcium, cardiac troponin I, chlorine, complement-1q (C1q), C-reactive protein, creatinine (CR), creatine kinase (CK), creatine kinase isoenzymes (CK-MB), CK/CK-MB, direct bilirubin, distribution width, eosinophilic number, eosinophilic percentage, fibrinogen, hematocrit, globulin, glutamyl transpeptidase, hemoglobin, indirect bilirubin, international standard ratio, kalium, lactate dehydrogenase, lymphocyte number, lymphocyte percentage (LYMPH), mean corpuscular volume, mean corpuscular hemoglobin, mean corpuscular hemoglobin concentration, mean platelet volume, monocyte number, monocyte percentage, myoglobin, natrium, neutrophil number, neutrophil percentage, prealbumin, percentage of large platelets, platelet, platelet distribution width, platelet hematocrit, prothrombin time, red blood cell, RDW-CV, retinol binding protein, serum amyloid A protein, thrombin time, total bilirubin, total protein, uric acid, white blood cell, 5-nucleotide enzyme, *β*2-microglobulin

**Table 2:** Profile of data sets.

Model layers	Conservative treatment	Surgical treatment	
M1	192	334	
M2		Intestinal nonnecrosis	Intestinal necrosis
		235	99

**Table 3:** Importance score of the top 20 characteristics.

Top 10 characteristics	The importance of value
(1) Creatine kinase isoenzymes (CK-MB)	0.049424
(2) Serum amyloid A protein (SAA)	0.048963
(3) Creatinine (CR)	0.047484
(4) C-reactive protein (CRP)	0.038210
(5) Chlorine (Cl)	0.034116
(6) CK/CK-MB	0.030630
(7) Albumin (ALB)	0.025981
(8) Albumin to globulin ratio (A/G)	0.024438
(9) Fibrinogen (CFbg)	0.023145
(10) Complement-C1q (C1q)	0.020137
(11) Lymphocyte percentage (LYMPH %)	0.019215
(12) Neutrophil number (NEUT#)	0.019043
(13) Age	0.018901
(14) Lymphocyte number (LYMPH#)	0.017077
(15) Total bilirubin (TBIL)	0.016674
(16) Neutrophil percentage (NEUT %)	0.016298
(17) Direct bilirubin (DBIL)	0.016145
(18) Lactate dehydrogenase (LDH)	0.016123
(19) Platelet (PLT)	0.016025
(20) Urea nitrogen (BUN)	0.015857

**Table 4:** Mean and variance of the top 10 features.

Feature	Positive sample		Negative sample	
	Mean	Variance	Mean	Variance
(1) Keratinase isoenzyme (CK-MB)	27.02	327.47	17.40	157.0
(2) Serum amyloid A (SAA)	80.71	11092.44	128.73	12152.6
(3) Creatinine (CR)	28.18	72.7	39.08	418.3
(4) C-reactive protein (CRP)	25.51	1916.3	65.32	6991.6
(5) Chlorine (Cl)	102.90	24.3	98.76	77.1
(6) CK/CK-MB	4.53	8.78	7.02	37.1
(7) Albumin (ALB)	44.85	25.5	42.01	51.1
(8) White ball ratio (A/G)	2.27	4.42	1.81	0.23
(9) Fibrinogen (Cob)	2.67	2.75	3.78	7.4
(10) Complement C1q (C1q)	179.54	2598.2	176.05	1505.5

**Table 5:** Fivefold results of the two-layer model.

	Fold	Acc (%)	Se (%)	Sp (%)	MCC
M1	1	80.19	68.85	89.23	0.57
	2	79.05	68.42	85.07	0.54
	3	78.10	78.38	77.94	0.55
	4	77.14	52.50	92.31	0.51
	5	85.71	72.22	92.75	0.68
	Mean	80.04	67.48	87.46	0.57
M2	Mean	66.78	13.16	90.15	0.18

**Table 6:** Accuracy and AUC of each model in the first layer.

Decision category	DNN	RF	SVM
Conservative patient	0.67	0.61	0.52
Patients undergoing surgery	0.87	0.92	0.79
Comprehensive accuracy	0.80	0.82	0.64
AUC	0.89	0.93	0.84

## Data Availability

The data used to support the findings of this study are included within the supplementary information file(s).

## Supplementary Materials

Supplementary Materials S1: benchmark dataset used for this study. There are 63 blood routine and liver and kidney function tests of 526 children with ileus in the supplementary file. In this study, the patient age values are converted to real numbers, and Labels 1, 2, and 3 are used to label samples according to whether they need to undergo surgery and whether their intestines are necrotic. The vacant value in the dataset is the problem in the source data, and the vacant value data has been fitted with methods mentioned in the paper.
